# Risk factors for prolonged preoperative waiting time of intertrochanteric fracture patients undergoing operative treatment

**DOI:** 10.1186/s12891-022-05865-x

**Published:** 2022-10-13

**Authors:** Fei Liu, Wen-jie Chang, Xu Wang, Rui Gong, Dao-tong Yuan, Yong-kui Zhang, Wen-peng Xie

**Affiliations:** 1 First Clinical College, Shandong University of Traditional Chinese Medicine, Jinan, China; 2 Department of Orthopedic Surgery, Affiliated Hospital of Shandong University of Traditional Chinese Medicine, 16369 Jingshi Road, Lixia District, 250014 Jinan, Shandong China; 3 Shandong Fupai Pharmaceutical Co., Ltd, Jinan, Shandong China

**Keywords:** Intertrochanteric fracture, Preoperative waiting time, Preoperative medical comorbidities, Deep vein thrombosis (DVT), Case characteristics

## Abstract

**Purpose:**

Intertrochanteric fracture is a common fracture in older adults. We observed the case characteristics of intertrochanteric fracture and analyzed the risk factors for prolonged preoperative waiting time based on patient data from a 6 year period. Investigate the post-admission treatment of intertrochanteric fracture.

**Methods:**

We retrospectively reviewed the medical records from July 2015 to July 2021 of patients hospitalized for intertrochanteric fracture who had undergone internal fixation surgery in the orthopedic ward of the Affiliated Hospital of Shandong University of Traditional Chinese Medicine. Data regarding gender, age, AO/OTA classification, preoperative waiting time, preoperative medical comorbidities, and complicated deep venous thrombosis (DVT) of lower limbs were collected. Statistical tests were used to evaluate the factors influencing preoperative preparation time and DVT.

**Results:**

A total of 1812 cases were retrospectively analyzed, 1258 patients (69.43%) had three or more medical comorbidities. The average preoperative waiting time was 5.09 ± 3.27 days. Advanced age, more preoperative medical comorbidities and DVT led to longer preoperative waiting times, and preoperative medical comorbidities were an independent risk factor. Patients with advanced age and preoperative medical comorbidities were more likely to have DVT.

**Conclusion:**

Age and preoperative medical comorbidities are risk factors for DVT and prolonged preoperative preparation time in intertrochanteric fracture patients. Preoperative medical comorbidities are an independent risk factors affecting the preoperative waiting time, and a combination of multiple comorbidities almost predicts the delay of the operation time.

## Introduction

Intertrochanteric fracture is often referred to as “the last fracture of life”, characterized by high age of onset, multiple risk factors, serious complications and high associated mortality. Epidemiological studies have led to the prediction that the number of hip fractures will reach 4.5 million to 6.3 million by 2050 as the world population ages [1, 2]. Rates of hip fractures are rising, particularly in many Asian countries [3]. Because intertrochanteric fractures account for 36 to 50% of hip fractures [4–6], these demographic changes have put enormous pressure on medical expenses and social costs throughout the world [1, 7].

Regarding the treatment of intertrochanteric fractures, a controversy exists concerning the coordination of preoperative waiting time. Elderly patients whose status is complicated with medical comorbidities need multidisciplinary comprehensive evaluation [8], especially to exclude circulatory, respiratory, and nervous system contraindications for surgery. On the other hand, performing surgery as early as possible is optimal in order to reduce bed rest and complications [9]. The time from admission to operation, thus, is an important concern of both researchers and patients. The preoperative waiting time in most hospitals in China is presently too long, and this issue is also evident in our hospital. Therefore, in this study, we analyzed the factors that prolong the preoperative preparation time and the characteristics of perioperative cases, and we analyzed the preparation procedures for patients with intertrochanteric fracture.

## Patients and methods

1. 2.1 Patient data collection: Data of 1845 patients with intertrochanteric fractures hospitalized for surgical treatment in the orthopedic ward of The Affiliated Hospital of Shandong University of Traditional Chinese Medicine from July 2015 to July 2021 were collected. Clinical data collected included gender, age, AO/OTA fracture classification, preoperative waiting time, preoperative medical comorbidities, and incidences of deep vein thrombosis (DVT).
2. 2.2 Subject criteria: Criteria for inclusion were (1) unilateral closed intertrochanteric fracture of femur was diagnosed by X-ray or CT examination; (2) age ≥ 18 years; (3) time from fracture to hospitalization ≤ 2 weeks; and (4) internal fixation used as the surgical method. Exclusion criteria were (1) conservative treatment or arthroplasty surgery used as the treatment method; (2) inability to move the affected limb normally before the injury; (3) multiple fractures of lower limbs; (4) pathological fracture; (5) surgical contraindications; or (6) incomplete data. A total of 1812 cases met the inclusion criteria, and 33 cases were excluded. This study was approved by the Ethics Committee of the Affiliated Hospital of Shandong University of Traditional Chinese Medicine.
3. 2.3 Clinical data collection: Data regarding gender, age and pre-operative preparation time were collected. Fracture classification of all patients was performed by two physicians with reference to the AO/OTA Fracture and Dislocation Classification [10]. If the two physicians disagreed on the fracture typing, a third senior orthopedic trauma surgeon assisted in the determination. All patients received Doppler ultrasound of both lower limbs before surgery; both the guidelines for the prevention of venous thromboembolism in Chinese orthopedic surgery and the international diagnostic standards for early DVT [11] use dual-power Doppler ultrasound as the first-line examination standard for DVT. Comorbidity data was collected from relevant ICD-10 codes [12] in the medical records that were assigned during the preoperative diagnosis, and these codes mainly were associated with cardiac disease, cerebrovascular disease, chronic lung disease, diabetes, renal disease, liver disease, gastrointestinal disease and hematological and neoplastic disorders.
4. 2.4 Statistical analysis: SPSS26.0 (IBM Corp, Armonk, NY, USA) software was used for statistical analysis. Quantitative data were analyzed by T-tests and one-way analysis of variance and multivariate analysis of variance tests, and counting data were statistically analyzed by χ2 tests and Cox regression analyses. The confidence interval was set at 95%, and *P* < 0.05 was considered statistically significant.

## Results

### **Patient clinical and demographic information**

A total of 1845 candidate patients were identified, and 1812 patients meeting the criteria were selected. Of the cases, 930 (51.3%) involved fractures of the right hip, and 882 involved fractures of the left hip. The mean preoperative waiting time was 5.09 ± 3.27days.

**Fig. 1:**
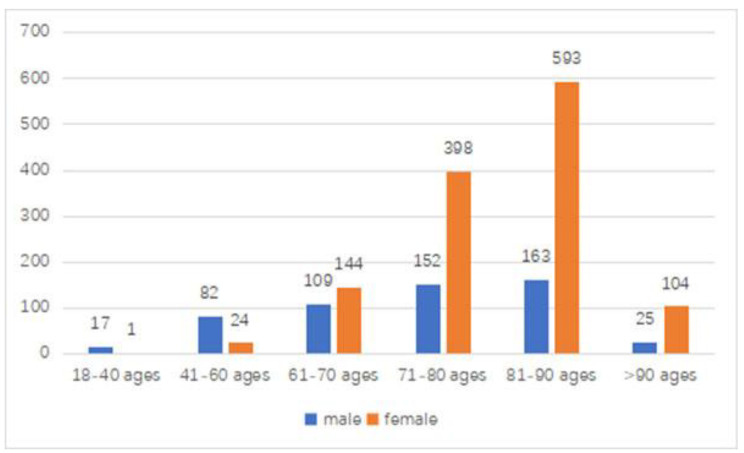
Numbers of intertrochanteric fractures of all ages

The subjects ranged in age from 18 to 100 years old, with an average age of 77.90 ± 11.00 years old. There were 548 males, with an average age of 72.74 ± 14.04 years, and the 1262 female subjects had an average age of 80.14 ± 8.46 years. The ratio of males to females was 1:2.31. The number of patients included in each age group is shown in Fig. 1 (Number of intertrochanteric fractures of all ages). The average age of the female patients was significantly higher than that of male patients (*P* < 0.001).

### **Fracture classification**

**Fig. 2:**
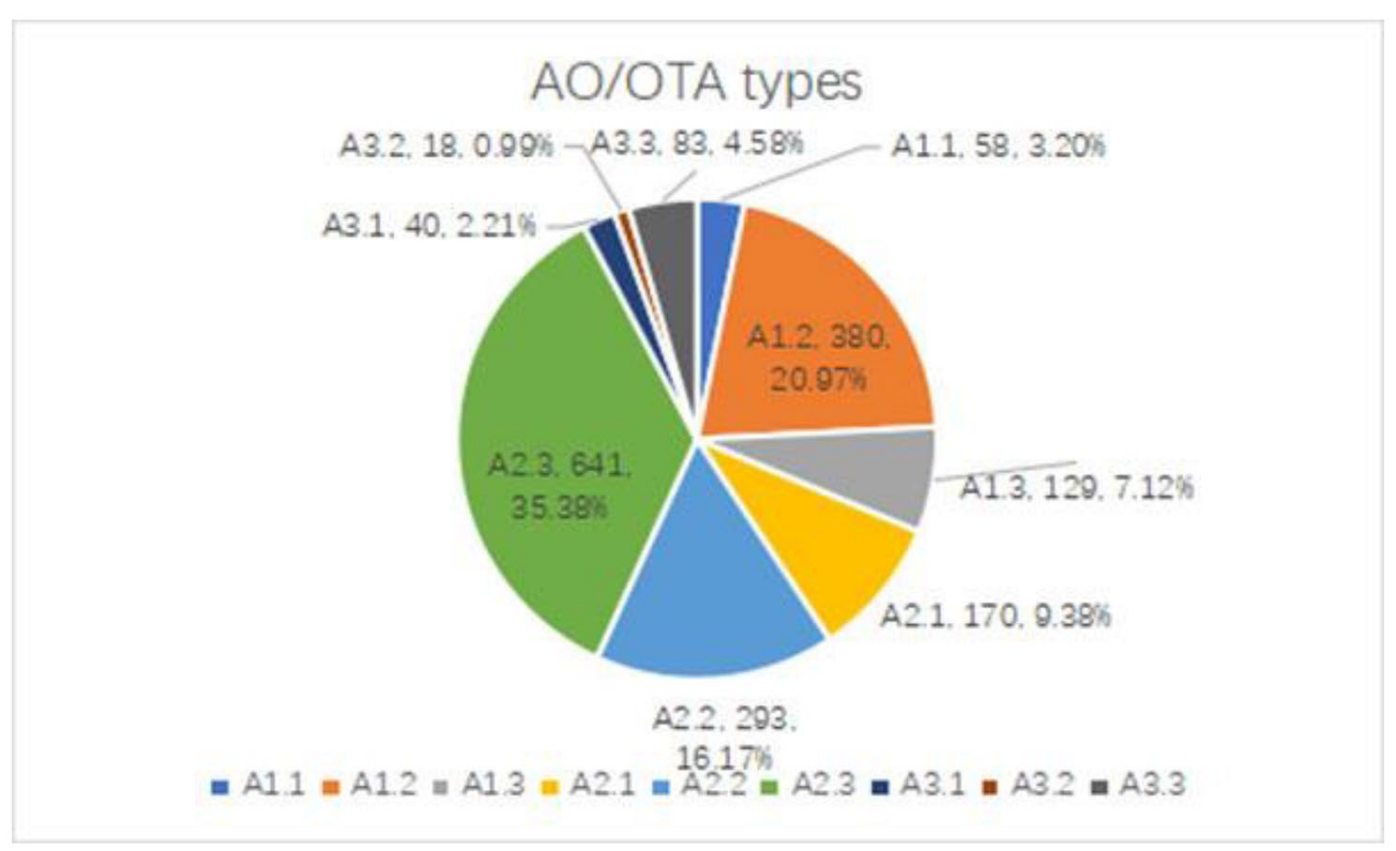
Proportion of fracture types

The imaging results from all patients were analyzed, and AO/OTA classification was performed. According to this analysis, the A2 fracture type was found in the largest number of patients; type A2 fractures accounted for 60.93% of the cases. Specifically, the A2.3 fracture type accounted for 35.38% of the overall number (Fig. 2. Proportion of fracture types).

### Medical comorbidities, including lower extremity DVT

As shown in Table 1, the cases of 1258 (69.43%) patients were complicated with three or more medical comorbidities. Preoperative examination found that 339 patients had lower extremity DVT, with an incidence of 18.71%, and no fatal pulmonary embolism occurred in any patients. Further analysis showed that advanced age was a risk factor for lower extremity DVT, and patients older than 70 years had a significantly increased risk of thrombosis(*P* < 0.001). The incidence of DVT was higher in patients with medical comorbidities than without medical comorbidities (Table 1).

**Table 1:** Analysis of the related factors of lower extremity deep vein thrombosis

	DTV patients(%)	Without DTV		χ2	P
sex				0.974	0.324
male	95(17.34%)		244		
female	244(19.30%)		1020		
age groups				14.336	**0.014**
18–40	2(11.11%)		16		
41–60	12(11.32%)		94		
61–70	32(12.65%)		224		
71–80	119(21.64%)		431		
81–90	150(19.84%)		606		
>91	24(18.60%)		105		
comorbidity				21.796	**<0.001**
0	3(2.77%)		105		
1	23(17.97%)		105		
2	54(16.88%)		266		
≥ 3	259(20.62%)		997		

Note: P < 0.05 is statistical significance

### **Preoperative preparation time**

The preoperative waiting time of intertrochanteric fracture patients from our hospital that were included in this study was 5.09 ± 3.27days. One-way ANOVA showed that age, AO/OTA classification, number of complicating medical comorbidities, and DVT were the influencing factors for the prolongation of preoperative waiting time. Multivariate ANOVA showed that number of complicating medical comorbidities was an independent risk factor affecting the preoperative waiting time (Table 2). The results of a Cox regression analysis excluded AO type as a factor affecting preoperative preparation time (Table 3).

**Table 2:** Analysis of variance of related factors of preoperative preparation time and related factors

		One-way Analysis of Variance		Multivariate Analysis of Variance	
factors		$\bar X$±S	P	F	P
sex			0.550	0.033	0.857
	male	4.95 ± 3.371			
	female	5.15 ± 3.321			
ages			**<0.001**	0.736	0.596
	18–40	3.28 ± 1.934			
	41–60	3.73 ± 3.276			
	61–70	4.56 ± 3.239			
	71–80	5.15 ± 3.274			
	81–90	5.43 ± 3.330			
	<90	5.09 ± 3.274			
AO types			**<0.001**	1.139	0.320
	A1	4.60 ± 2.811			
	A2	5.30 ± 3.423			
	A3	5.41 ± 3.275			
comorbidity			**<0.001**	10.031	**<0.001**
	0	2.10 ± 1.199			
	1	3.19 ± 1.724			
	2	3.86 ± 1.782			
	≥ 3	5.86 ± 3.483			
DVT			**<0.001**	0.000	0.990
	yes	6.25 ± 3.840			
	no	4.82 ± 3.070			

Note: P < 0.05 is statistical significance

**Table 3:** Analysis of multivariate Cox regression of preoperative preparation time and related factors

factors	β	SE	Wald	P	Exp(β)	95% CI	
sex (male vs. female)	-0.78	0.055	2.039	0.153	0.925	0.830	1.030
ages	0.011	0.003	15.161	**<0.001**	1.011	1.005	1.016
AO types			4.396	0.111			
A2 vs. A1	-0.076	0.053	2.111	0.146	0.926	0.835	1.027
A3 vs. A1	-0.185	0.095	3.759	0.053	0.831	0.690	1.002
comorbidity			340.849	**<0.001**			
1 vs. 0	0.912	0.137	44.156	**<0.001**	0.402	0.307	0.526
2 vs. 0	1.311	0.125	110.841	**<0.001**	0.271	0.212	0.345
≥ 3 vs. 0	2.082	0.128	264.728	**<0.001**	0.125	0.097	0.160
DVT	-0.296	0.061	23.362	**<0.001**	0.744	0.660	0.839

Note: P < 0.05 is statistical significance

## Discussion

Our study findings regarding the impact of demographic factors on fracture incidence were similar to previous studies that have suggested that 90% of intertrochanteric fractures occur in people over 50 years of age, and that these fractures tend to occur more frequently in middle-aged and older women than in men. The connections of fracture frequency with sex and age are likely explained in part by a drop in levels of estrogen in women after menopause, leading to more rapid bone loss [13]. The World Health Organization reports that the risk of osteoporotic fractures increases 2.6 times with a decrease in bone density of more than one standard deviation from the mean [14]. The number of falls is also reported to be a risk factor for intertrochanteric fractures, and women tend to fall approximately twice as often as men [15]. Lim et al. [16] found that due to the different distribution of skin, fat, muscle, tendon and fascia layers in each area of the buttock, the soft tissue in the posterior area of the hip is less hard, and these factors can make hip fractures more likely upon falling from standing or when falling out of bed.

Most of the subjects included in this study were elderly patients whose cases were complicated with medical comorbidities. The proportion of patients aged over 70 years was 86.43%, and our data showed that 94.04% of patients had at least one medical comorbidity prior to surgery, while 69.43% of patients had three or more medical comorbidities. In particular, the incidence of DVT among the intertrochanteric fractures patients was 18.71%. Virchow’s triad describes the three main causes of DVT, namely stasis, hypercoagulability, and endothelial changes [17]. Injury to the vascular endothelium as a direct result of a fracture can overactivate the clotting system and accelerate thrombosis [18]. Age, diabetes, and elevated D-dimer levels have been found to be potential risk factors and indicators for preoperative DVT in patients with lower extremity fractures [19]. Our analysis demonstrated that the incidence of lower limb DVT was higher in patients with medical comorbidities than in patients without medical comorbidities. Analysis under Bonferroni correction showed no significant variability in the number of comorbid medical conditions.

The scientific consensus regarding care for intertrochanteric fractures is that they should be operated on within 48 h of the initial injury [20]. However, in our center, the emergency surgery of hip fracture is rare, with only a small portion of hip fractures being treated surgically within 48 h. This relatively slow move to surgery occurs for multiple reasons. First of all, China is a developing nation and tends to be a late adopter of all medical procedures; therefore, the level of care needs to be raised. In addition, because our hospital is a medium-sized medical facility, the lengthy preoperative examination was significant variable affecting the preoperative waiting time. Clinically complex evaluation by physicians and anesthesiologists, as well as conservative treatment approaches extend consultation time and delay the surgery. There are also objective reasons, such as the use of anticoagulant drugs causes an inevitable delay in anesthesia. According to our analysis, concomitant medical comorbidities are also independent risk factors affecting the preoperative waiting time. In particular, only 12.5% patients with three or more comorbidities receive surgery in the same time than healthy patients, an alarming statistic warrants attention. Age and DVT are additional factors that need to be evaluated in a timely manner due to their important impacts on preoperative preparation time.

Early surgery for hip fractures has been shown to benefit older patients, even if they have preoperative comorbidities or take other medications [9]. Accordingly, operation within 48 h of initial injury is the treatment target in most countries [21–23]. Those who have complicating conditions should have as short of a preoperative waiting period as possible, delays of more than 24 h will lead to an increased risk of patient mortality [24]. On the other hand, other studies have shown that while a waiting time of more than 48 h before surgery leads to a significant increase in postoperative mortality in otherwise healthy patients, a 48 h wait actually has a protective effect on patients with serious medical complications [25, 26]. Pincus, et al. [27] point out that the patients taking direct oral anticoagulants benefit from an increased preoperative waiting time and that an increased preoperative waiting time was associated with a reduction in mortality related to hip fracture. Others have explained that the key delay period with regard to increased mortality is the delay from injury to hospitalization, not the delay from hospitalization to surgery [28]. Patients with electrolyte imbalances and anemia can typically be treated optimally without delaying surgery. While it must be adjusted before surgery if plasma sodium < 120 or > 150 mmol/L and potassium < 2.8 or > 6.0 mmol/L, these are valid justifications for postponing surgery [29].

Based on our experience, it is optimal to utilize the emergency green channel integrated management strategy for the preoperative examination of patients with hip fracture. This strategy includes the rapid performing of echocardiography as an emergency examination, to prevent additional surgical delay [30]. The need for emergency surgery should be evaluated according to the Charlson Comorbidity Index score and American Society of Anaesthesiology score(ASA), and analgesics showed be used actively [29]. A multidisciplinary approach can reduce postoperative complications, length of hospital stay and mortality [31]. Clinicians need to learn and accept new management concepts, such as the evaluation of the perioperative risk of hip fracture in the elderly with diabetes [32]. It is necessary to analyze which treatments must be carried out before surgery, and make corresponding decisions based on clinical factors. It is more vital than ever to aggressively, consistently, and precisely manage medical comorbidities in older people, to stop the progression of the disease. Patients who have well-controlled medical comorbidities can have surgery sooner following an unanticipated event.

Therefore, once patients with intertrochanteric fracture are admitted, clinical variables should be used to decide if surgery needs can be done immediately or wait. Patients with normal organ and system functions or those who fall within the compensatory range (ASA III or below) can get emergency surgery after DVT has been ruled out. The rate of postponed surgery is significant if the comorbidities are under inadequate medical care.

## Limitations

Our study was accompanied by several limitations. The fact that this was a single-centre study may have led to some bias in the data. In addition, some subjective reasons for prolonged pre-operative preparation may have impacted the analysis. Finally, many patients were lost to follow-up after surgery, and we were not able to analyze the influence of preoperative waiting time on the postoperative recovery and mortality.

## Conclusion

Age and preoperative medical comorbidities are risk factors for DVT and prolonged preoperative preparation time in intertrochanteric fracture patients. Preoperative medical comorbidities are an independent risk factors affecting the preoperative waiting time, and a combination of multiple comorbidities almost predicts the delay of the operation time. It is more vital than ever to aggressively, consistently, and precisely manage medical comorbidities in older people.

## Data Availability

The datasets used and/or review during the current study are available from the corresponding author on reasonable request.

## Abbreviations

DVT: Deep venous thrombosis
